# Glycemia, Insulin Sensitivity, and Secretion Improve 3 Months Post-sleeve Gastrectomy in Youth With Type 2 Diabetes

**DOI:** 10.1210/jendso/bvaf020

**Published:** 2025-01-28

**Authors:** Tyler J Dobbs, Melanie G Cree, Alex J Bailey, Amy D Baumgartner, Justin Garrish, Cecelia Diniz-Behn, Laura Pyle, Megan M Kelsey, Amy S Shah, Thomas H Inge, Petter Bjornstad, Kristen J Nadeau

**Affiliations:** University of Colorado Anschutz Medical Campus and Children's Hospital Colorado, Aurora, CO 80045, USA; University of Colorado Anschutz Medical Campus and Children's Hospital Colorado, Aurora, CO 80045, USA; University of Colorado Anschutz Medical Campus and Children's Hospital Colorado, Aurora, CO 80045, USA; University of Colorado Anschutz Medical Campus and Children's Hospital Colorado, Aurora, CO 80045, USA; Colorado School of Mines, Golden, CO 80401, USA; Colorado School of Mines, Golden, CO 80401, USA; University of Colorado Anschutz Medical Campus and Children's Hospital Colorado, Aurora, CO 80045, USA; University of Colorado Anschutz Medical Campus and Children's Hospital Colorado, Aurora, CO 80045, USA; The University of Cincinnati and Cincinnati Children's Hospital Medical Center, Cincinnati, OH 45229, USA; Northwestern University and Lurie Children's Hospital of Chicago, Chicago, IL 60611, USA; University of Colorado Anschutz Medical Campus and Children's Hospital Colorado, Aurora, CO 80045, USA; University of Washington and Seattle Children's Hospital, Seattle, WA 98105, USA; University of Colorado Anschutz Medical Campus and Children's Hospital Colorado, Aurora, CO 80045, USA

**Keywords:** metabolic bariatric surgery, youth-onset type 2 diabetes, incretin, vertical sleeve gastrectomy

## Abstract

**Context:**

Metabolic bariatric surgery reduces weight in youth with severe obesity; however, its impacts on youth-onset type 2 diabetes (T2D) are unclear.

**Objective:**

We evaluated short-term outcomes in youth with T2D 3 months after vertical sleeve gastrectomy (VSG).

**Design:**

Longitudinal, observational study in the Impact of Metabolic surgery on Pancreatic, Renal, and cardiOVascular hEalth in youth with T2D study (IMPROVE-T2D).

**Setting:**

Academic medical university and children's hospital.

**Participants:**

Fourteen youth with T2D [mean age ± SD 16.8 ± 1.4 years; 50% female, pre-VSG hemoglobin A1c (HbA1c) 6.6 ± 0.2%; diabetes duration 17.6 ± 13.8 months; age at diabetes diagnosis 15.9 ± 1.4 years; body mass index (BMI) 46.7 ± 2 kg/m^2^].

**Interventions:**

Participants underwent a mixed-meal tolerance test (MMTT), body composition, and indirect calorimetry before and 3 months after VSG.

**Main Outcomes:**

Glycemic control (HbA1c, diabetes medications), insulin sensitivity (Matsuda Index, Homeostasis Model of Insulin Sensitivity, oral minimal model), and secretion (C-peptide model).

**Results:**

After VSG, weight and BMI decreased (25.2 ± 5.6 kg [19%], −8.7 ± 2 kg/m^2^ [18%], respectively, *P* < .001). Body fat decreased (4.5%, *P* = .012), with reductions of 14.1 ± 5.4 kg of fat mass (*P* = .005) and 4.5 kg of fat-free mass (*P* = .034). HbA1c decreased from 6.6 ± 0.2% to 5.7 ± 0.2% (*P* = .003), with 86% of participants no longer requiring diabetes medications. Glucose was lower throughout the MMTT, with insulin, C-peptide, free fatty acids, glucagon-like peptide-1, and peptide-YY significantly changing postsurgery (*P* < .05 for all). Insulin sensitivity and insulin secretion rate during the MMTT significantly improved.

**Conclusion:**

Three months post-VSG, youth showed significant improvements in weight, body composition, insulin sensitivity and secretion, and glycemic control, with most no longer requiring diabetes medications.

Obesity remains a major health problem affecting 20.6% of youth [1], with rates of youth-onset type 2 diabetes and associated complications [2] projected to increase 700% by 2060 [3]. Youth-onset type 2 diabetes has many unique features distinguishing it from adult onset and appears more severe than in adults, with youth having markedly lower insulin sensitivity (SI); fasting and stimulated insulin and C-peptide secretion that is approximately twice as high; lower stimulated glucagon and glucagon-like peptide-1 (GLP-1) secretion; less response to diabetes medications and lifestyle interventions regarding weight loss; β-cell function and glycemic control; and more rapid onset of pancreatic β-cell failure, cardiovascular, and kidney disease [4-8]. In the Treatment Options for type 2 diabetes in Youth (TODAY) study, 45.6% of youth progressed to glycemic failure by 3.9 years after diagnosis, with a median time to failure of only 11.5 months despite short diabetes duration (7.8 months) [9]. Importantly, the metformin failure rate was 51.7%, approximately 2.5x higher than the metformin failure rate reported in adults with type 2 diabetes [10]. Similarly, the metformin plus rosiglitazone failure rates in TODAY were approximately 2x higher than reported in adults, and the degree of β-cell function decline was ∼2 to 4x higher in youth vs adults [10]. Moreover, in the Restoring Insulin Secretion study, while 12 months of metformin or 3 months of insulin glargine followed by 9 months of metformin slowed β-cell failure in adults with prediabetes and type 2 diabetes, β-cell failure continued in the identically treated youth, findings that would support more aggressive treatment approaches for youth-onset type 2 diabetes [11].

The recent American Academy of Pediatrics guidelines recommend nutritional, physical activity, behavioral, and obesity pharmacotherapy for adolescents ≥12 years old with obesity [body mass index (BMI) ≥95th percentile] and evaluation for metabolic bariatric surgery (MBS) for adolescents ≥13 years old with class II obesity (BMI ≥120% of the 95th percentile) plus a major comorbidity or with class III obesity (BMI >140% of the 95th percentile) either with or without a documented comorbidity [12, 13]. MBS results in significant and durable weight loss of nearly 30% after 7 years in adults [14], with short- and medium-term metabolic effects that appear independent of weight loss, including improvements in glycemia, hypertension, and hyperlipidemia [15] and diabetes remission in most adults [16]. However, applying these data to youth is challenging given the severe insulin resistance of puberty, marked compensatory hyperinsulinemia, and early and rapid β-cell failure.

Roux-en-Y gastric bypass (RYGB) in adolescents results in similar weight loss as in adults, with about 27% absolute weight loss at 5 years in both the Adolescent Morbid Obesity Surgery study [17] and the Teen Longitudinal Assessment of Bariatric Surgery (Teen-LABS) study, but few adolescent data exist on the impact of MBS on glycemic control, SI, or insulin secretion. The limited data in youth with type 2 diabetes from the Teen-LABS study support the benefits of MBS in adolescents regarding glycemic control, with 95% experiencing diabetes remission at 3 years post-MBS [18]. However, Teen-LABS studied mostly youth without diabetes, the majority were Non-Hispanic White, and a majority underwent RYGB. Vertical sleeve gastrectomy (VSG) is now the most common MBS procedure performed in youth and adults due at least in part to its improved safety profile [19]. Moreover, most MBS studies lack short-term outcomes, missing valuable opportunities to examine the early effect of MBS before full weight loss is achieved on the glycemic, metabolic, and hormonal response to feeding, including potential weight loss independent improvements in health. Given the well-established phenotypic differences between youth and adults with type 2 diabetes, such as more severe hyperinsulinemia and poorer response to medical treatments in youth, it is reasonable to hypothesize that youth with type 2 diabetes may respond differently to MBS. Therefore, the aim of the Impact of Metabolic surgery on Pancreatic, Renal, and cardiOVascular hEalth in youth with type 2 diabetes (IMPROVE-T2D) study was to determine the early effect of VSG on glycemia (primary outcome), SI, insulin secretion, diabetes medication use, substrate oxidation, and the incretin response to feeding in youth-onset type 2 diabetes before and 3 months after undergoing VSG.

## Materials and Methods

### Participants

Fourteen adolescents ages 14 to 19 years with confirmed antibody-negative youth-onset type 2 diabetes (negative for insulin, glutamic acid decarboxylase, islet-cell, and zinc transporter antibodies) and severe obesity, scheduled for VSG were recruited from the pediatric MBS clinic at Children's Hospital Colorado. The study was approved by the Colorado Multiple Institutional Review Board. Written informed consent was obtained by qualified research personnel for participants ages 18 years or older and assent for participants below 18 years of age in addition to the parent(s) or legal guardian providing consent.

Screening included a medical history and physical exam including pubertal Tanner staging by the study physician. Exclusion criteria included diabetes diagnosis after age 18, prepubertal status, anemia, pregnancy or breastfeeding, or recent diabetic ketoacidosis. Participants were asked to refrain from strenuous physical activity for 3 days prior to study visits to limit the impact of the last bout of acute exercise on SI and metabolism. Metformin and sodium-glucose cotransporter-2 inhibitors (SGLT-2i) were discontinued 72 hours, GLP-1 receptor analogs (GLP-1RA) 1 week, and long-acting insulin 24 hours prior to their study visit if applicable. Three participants were on GLP-1RA agents (1 on daily liraglutide, half-life approximately 13 hours, and 2 on daily semaglutide oral tablets, half-life approximately 1 week) Participants arrived at the pediatric research center fasting in the AM to undergo all testing.

### Surgical and Medication Management

VSG was performed under general anesthesia with the patient in supine position. Pneumoperitoneum was established using CO_2_, and 5 small abdominal incisions were made to insert trocars and laparoscopic instruments. A 38F calibration bougie was introduced orally into the stomach to guide the resection. Using a linear stapler, ∼75% to 80% of the stomach was vertically resected, starting from the antrum (∼6 cm from the distal pylorus) up to the angle of His, creating a tubular gastric pouch. The resected stomach portion was removed through the umbilical trocar site. Postoperatively, patients were monitored and discharged within 1 to 2 days off of diabetes medications, and the patient's metformin was restarted if home glucometer readings rose to ≥126 mg/dL fasting or ≥200 mg/dL postprandially. Female participants received the standard contraceptive clinical care at our institution, which involves being offered long-acting reversible contraceptives in the form of intrauterine devices or implantable progesterone-only devices at the time of surgery if not currently on birth control. All female participants were either on an intrauterine device or implantable progesterone-only device after surgery.

After VSG, our adolescent MBS clinic follows a standard diet stage progression [20] to address nutrient needs, beginning with sugar-free clear liquids postoperatively on days 1 to 2, followed by full high protein liquids for the following 2 weeks. Soft protein foods are then introduced by weeks 3 and 4. By 2 to 3 months postoperatively, patients typically return to a high-protein, solid-food diet.

### Anthropometrics

Height was measured to the nearest 0.1 cm on a standard stadiometer and body weight to the nearest 0.1 kg on a digital scale. BMI and BMI percentiles were calculated. Waist and hip circumference were measured using a flexible measuring tape to the nearest 0.1 cm. Waist circumference was measured at the narrowest part of the waist above the umbilicus and below the xiphoid process. Hip circumference was measured at the maximal girth of the hip, around the buttocks above the gluteal fold. Body composition was measured via air-displacement plethysmography (BodPod, Cosmed Inc., Rome, Italy) to determine fat mass and fat-free mass (FFM) using standard methods [21]. Systolic and diastolic blood pressure were measured twice manually after 5 minutes of rest using a stethoscope and sphygmomanometer and the average of the 2 readings was used. Pulse was manually counted for 60 seconds.

### Mixed-meal Tolerance Test and Indirect Calorimetry

A modified mixed-meal tolerance test (MMTT) with indirect calorimetry was performed using Boost + (Nestle Corp., Switzerland, 45 g glucose, 14 g fat, 14 g protein). To prevent potential gastrointestinal distress in a smaller-volume stomach postoperatively, we modified the MMTT at both pre- and postsurgical visits by dividing the Boost + into 7 1.1 oz aliquots consumed every 5 minutes for 30 minutes, starting at time 0. Blood was collected for glucose and insulin at time points −10, 0, 10, 20, 30, 45, 60, 90, 120, 150,180, and 240 minutes; C-peptide at −10, 0, 10, 20, 30, 60, 90, 120,180, and 240 minutes; GLP-1 and glucagon at −10, 0, 10, 30, 45, 60, 120, and 240 minutes; free-fatty acids (FFA) and peptide YY(PYY) at −10, 0, 30, 60, 120, 180, and 240 minutes.

Indirect calorimetry via a metabolic cart (CareFusion, CA) was used to measure resting energy expenditure (REE), carbohydrate and fat oxidation, and metabolic flexibility with feeding. The cart was calibrated to manufacturer specifications prior to each measurement and indirect calorimetry measured just prior to the MMTT start and again 60 minutes after beginning Boost + consumption. Oxygen consumption (VO_2_) and carbon dioxide production (VCO_2_) in both absolute (L/min) and relative (mL/kg/min) amounts were collected to determine REE using the Weir equation [22], respiratory quotient (RQ) (CO_2_ produced/O_2_ consumed), and rates of carbohydrate vs fat oxidation using previously established methods [23]. Identical measures were taken at baseline and 60 minutes after the MMTT start to calculate change in carbohydrate and fat oxidation and metabolic flexibility. Participants were laid supine in a dimly lit and quiet room and respiratory gas exchange measured within a canopy hood circuit for at least 20 minutes until steady state was achieved. Measurements were included if at least 15 minutes of steady state was met with less than 10% fluctuation in minute ventilation and O_2_ consumption and less than 5% fluctuation in RQ [24].

### Measures of Insulin Sensitivity and Secretion

The Homeostasis Model of Insulin Sensitivity (HOMA-IR) was used to assess fasting SI [25] the Matsuda Index to assess SI, and the disposition index to assess β-cell function (the product of Matsuda Index and insulinogenic index) during the MMTT [26]. The Oral Minimal Model (OMM) was also used to model glucose dynamics and estimate SI during the MMTT, after modification for use in adolescents with insulin resistance and in the MMTT setting [27]. OMM outcomes are briefly described later, with detailed formulas available in Table 1.

**Table 1.** Oral minimal model calculations

Outcome	Equation	Variable definitions
Oral minimal model	$\dot{G}(t)=-[S_{G}+X(t)]G(t)+p_{1}G_{b}+\frac{1}{V}Ra(\alpha ,\;t)$ , $G(0)=G_{b},$ $\dot{X}(t)=-P_{2}X(t)+p_{3}\max(I(t)-I_{b},\;0)$, X(0)=0,	G(t) is glucose concentration; X(t) is insulin action; I(t) is insulin concentration; parameters G_b_ and I_b_ are basal glucose and insulin concentrations, respectively; S_G_, p2, and p3 are rate parameters; *V* is the volume of glucose distribution, and Ra(α,t) is a piecewise-linear function describing the rate of appearance of exogenous glucose
Insulin sensitivity (Static SI)	$S_{I}=(p_{3}/p_{2})\cdot V$ dl/kg/min per mU/mL	p2 and p3 are rate parameters; *V* is the volume of glucose distribution
Fractional glucose effectiveness parameter ($S_{G}$)	$S_{G}=\frac{GEZI+S_{I}\cdot I_{b}}{V},$	GEZI represents glucose effectiveness at zero insulin and is fixed to 0.025 dL/kg/min presurgery when participants had a diagnosis of type 2 diabetes and 0.036 dL/kg/min postsurgery when type 2 diabetes had resolved [28]
Dynamic SI^D^	$S_{I}^{D}=S_{I}\left[1-\frac{1-\exp\;(-p_{2}T)}{\;p_{2}T}\right]$	p2 is a rate parameter, and parameter *T* is fixed to 60 minutes [29]
Efficiency	Efficiency=$\;S_{I}^{D}/S_{I}$	$S_{I}$ = static insulin sensitivity $S_{I}^{D}=\mathrm{dynamic}\;\mathrm{insulin}\;\mathrm{sensitivity}$
β-cell responsivity (Φ)	$\Phi =\frac{\int_{0}^{T}\;\max\{ISR(t)-ISR_{b},\;0\}dt}{\int_{0}^{T}\;max\{G(t)-G_{b},\;0\}\;dt},$	SR(t) and $ISR_{b}$ represent the ISR at time *t* and at basal, respectively, in pmol/L/min [30]

The OMM is a 1-compartment model to estimate SI from an oral glucose tolerance test or a mixed meal using a differential equations-based approach to describe glucose dynamics in response to an oral challenge [31]. OMM results from a mixed meal have been shown to correlate well with those from an oral glucose tolerance test [32, 33]. The OMM-derived indices, static SI and dynamic SI, capture the sensitivity to and rapidity of insulin action increases, respectively, in response to rises in glucose concentration and OMM efficiency as the ratio of dynamic to static SI.

Glucose concentrations for all participants returned to their respective baseline values within 4 hours (240 minutes). Therefore, we implemented a truncated form of OMM [27], with glucose rate of appearance (Ra) break points at 0, 10, 30, 60, 90, 120, 150, 180, and 240 minutes postmeal. The area under the Ra curve was constrained to reflect total glucose absorbed based on glucose dosage, participant body weight, and the assumption that 90% of ingested glucose is absorbed over 4 hours. As previously described [31], we fixed the distribution volume V=1.45 dL/kg. Since insulin concentrations may fall below basal values during the MMTT, we incorporated a maximum function in the equation so that only insulin concentrations above basal contribute to changing insulin action. Model equations were implemented in SAAM II (SAAM II software v 2.3, Nanomath).

### Inferring Insulin Secretion Rate to Estimate β-Cell Responsivity

For model-based quantification of β-cell responsivity, we first reconstructed the continuous insulin secretion rate (ISR) profile for each participant from MMTT C-peptide data using a C-peptide model adapted from our published Bayesian hierarchical model [34]. The original model describes the ISR normalized by the volume of distribution as a mixed-effects Gaussian process and maps the continuous (normalized) ISR to discretely sampled C-peptide measurements according to the C-peptide dynamics model of Eaton et al [35]. In the modified model, we instead work with the logarithm of ISR to enforce positivity. Rate parameters in the C-peptide model were estimated from participant age and type 2 diabetes status using the equations described by van Cauter and colleagues [36]. From the ISR profile, we estimated the molar concentration of insulin secreted over the first 30 (early-phase) minutes, the first 60 minutes, and the entire 4 hours as the area under the ISR profile divided by the respective time durations. We computed model-based β-cell responsivity as the ratio of early-phase insulin secretion, as estimated by the C-peptide model, to the area under the glucose profile, as estimated by OMM, as in [30], but we truncated the integrals at 30 minutes, rather than including the full 4 hours, to focus on early-phase insulin response. The model and model-based calculations were implemented in R version 4.0 (R Core Team, Vienna).

### Biochemical Assays

All laboratory testing was performed by standard methods at the Core Laboratory facility at the Colorado Clinical and Translational Sciences Research Center Laboratory. A comprehensive metabolic panel and lipid panel were analyzed using standard clinical methods. Glucose was analyzed with a portable, hospital-grade glucometer (Stat Strip, Nova Biomedical, Waltham, MA) by the glucose-oxidase method, hemoglobin A1C (HbA1c) by the potassium ferricyanide method (Siemens DCA Vantage), insulin by the chemiluminescent immunoassay method (Beckman Coulter, RRID: AB_2756878 https://scicrunch.org/resolver/AB_2756878), C-peptide and GLP-1 by ELISA (Mercodia, RRID: AB_2750847 https://scicrunch.org/resolver/AB_2750847 and AB_2892202 https://scicrunch.org/resolver/AB_2892202, respectively), glucagon and PYY by radioimmunoassay (Millipore, RRID: AB_2757819 https://scicrunch.org/resolver/AB_2757819 and AB_2801578 https://scicrunch.org/resolver/AB_2801578, respectively), and FFA by the enzymatic method (WaKo Chemicals, USA).

### Statistical Analysis

Participant characteristics were summarized as mean and SD or as counts and percentages. Repeated measures mixed-effects models with a compound-symmetric correlation structure were used to examine changes from baseline to month 3. Unadjusted models and models adjusted for weight loss were performed. Estimated marginal means were calculated at each visit. Similar mixed-effects models were used to test the difference between MMTT labs before and after surgery while accounting for the correlation of repeated measures within a participant. Estimated marginal means were calculated for each time point of the MMTT within a visit, and pairwise comparisons were used to test whether each time point differed across visits. A *P*-value of <.05 was considered statistically significant. A priori power analysis (G*Power version 3.1) was conducted to determine the minimum sample size required to achieve statistical power using previously published data [37]. A sample size of 10 participants was predicted to be needed to achieve 80% power to detect an effect size (f²) of 1.01 to detect statistically significant differences in the primary outcome of HbA1c using an F-test with a significance level (α) of 0.05. Analyses were performed using R version 4.0 (R Core Team, Vienna) and figures created using GraphPad Prism version 10.2.1 (GraphPad Software, Boston, MA).

## Results

### Demographics of Cohort and Clinical Outcomes Before and After Surgery

The 14 participants had a mean age of 16.8 ± 1.4 years and were 50% female; a majority were Hispanic White (78.6%) (Table 2). The average age at diagnosis of diabetes was 15.9 ± 1.4 years and average diabetes duration was 17.6 ± 13.8 months. All participants were tolerating a normal, solid-food diet by their 3-month study visit. VSG induced significant reductions in weight (25.2 kg, or a 19% decrease from presurgery), BMI, and BMI percentile (all *P* < .001). Body composition was improved after surgery with a 4.5% reduction in body fat (*P* = .012), resulting in an absolute reduction of 14.1 kg of fat mass (*P* = .005) and 4.5 kg of FFM (*P* = .034). Both waist and hip circumference were also significantly reduced after surgery (*P* < .001 and *P* = .001, respectively).

**Table 2.** Participant clinical characteristics pre- and postsurgery

Variable	Presurgery	Postsurgery	*P* (unadjusted)	*P* (adjusted)
Age (years)	16.8 ± 1.4			
Sex (M/F)	7/7			
Race/ethnicity [n (%)]				
Hispanic or Latino	11 (78.6)			
Non-Hispanic Black	2 (14.3)			
Non-Hispanic White	1 (7.2)			
Diabetes duration (months)	17.6 ± 13.8			
Age at diagnosis of diabetes (yr)	15.9 ± 1.4			
Height (cm)	169.5 ± 7	169.5 ± 7.3	.747	.747
Weight (kg)	134.4 ± 5.6	109.2 ± 5.6	<.001	
BMI (kg/m^2^)	46.7 ± 2	38 ± 2	<.001	
BMI percentile	161.3 ± 29	130.4 ± 21.1	<.001	
Waist circumference (cm)	133.6 ± 3.1	117.3 ± 3.1	<.001	<.001
Hip circumference (cm)	129.2 ± 2	117.4 ± 4	.001	.001
Body fat (%)	50.4 ± 1.4	45.9 ± 1.5	.012	.014
Fat mass (kg)	66.6 ± 5.1	52.5 ± 5.4	.005	.006
Fat-free mass (kg)	64.5 ± 3.5	60 ± 3.7	.034	.037
HbA1c (%)	6.6 ± 0.2	5.7 ± 0.2	.003	.003
Metformin use [n (%)]	14 (100%)	2 (14.3%)	n/a	
SGLT-2i use [n (%)]	5 (35.7%)	0 (0%)		
GLP-1RA use [n (%)]	3 (21.4%)	0 (0%)		
Insulin use [n (%)]	2 (14.3%)	0 (0%)		
Resting heart rate (bpm)	94 ± 5	74 ± 5	.018	.024
SBP (mmHg)	129 ± 4	123 ± 4	.087	.09
DBP (mmHg)	75 ± 2	68 ± 2	.075	.083
Total cholesterol (mg/dL)	180 ± 7	144 ± 7	<.001	<.001
LDL-C (mg/dL)	119 ± 8	97 ± 8	.002	.002
HDL-C (mg/dL)	34 ± 2	34 ± 2	.872	.872
Triglycerides (mg/dL)	271 ± 47	123 ± 47	.024	.024
ALT (U/L)	65 ± 7	29 ± 8	.002	.002
AST (U/L)	53 ± 5	34 ± 6	.046	.037

Continuous variables are shown as mean ± SE or n (%) and were tested using repeated measures mixed-effects models and are shown for both unadjusted and adjusting for percent weight loss at 3 months. Medications are shown as descriptive statistics. Significance was set at *P* < .05.

Abbreviations: ALT, alanine aminotransferase; AST, aspartate aminotransferase; BMI, body mass index; , DBP, diastolic blood pressure; GLP-1RA, glucagon-like peptide 1 receptor analog; HDL, high-density lipoprotein; LDL, low-density lipoprotein; SBP, systolic blood pressure; SGLT-2i, sodium-glucose cotransporter-2 inhibitor.

HbA1c decreased from 6.6%±0.2 to 5.7%±0.2 (*P* = .003). Notably, all participants were on at least 1 diabetes medication at baseline, with 70% of participants requiring multiple agents (6 participants on 1 medication, 6 on 2 medications, and 2 on 3 medications). Of these, all were on metformin, 5 (35.7%) were on an SGLT-2i, 3 (21.4%) were on a GLP-1RA, and 2 (14.3%) were on glargine insulin. After surgery, 12 participants (85.7%) no longer required diabetes medication, with a mean HbA1c in this subset of 5.5% (range 5.0-6.0%). Only 2 participants remained on diabetes medication, both on only metformin with an HbA1c of 6.3% and 6.6%, respectively. At baseline, these 2 participants (who continued to need diabetes medications at 3 months) were on metformin plus a SGLT-2i and metformin plus insulin; 1 participant was an African American male with a preoperative BMI of 60 kg/m^2^ and the other was a 19-year-old Hispanic female with a diabetes duration of 50 months. Although we were not powered to detect sex differences, there were no significant differences by sex in the primary outcomes of weight loss or HbA1c (data not shown).

Resting heart rate was significantly reduced from 94 ± 5 to 74 ± 5 bpm (*P* = .018). Significant improvements were observed in total cholesterol (*P* < .001), low-density lipoprotein cholesterol (*P* = .002), triglycerides (*P* = .024), alanine aminotransferase (*P* = .002), and aspartate aminotransferase (*P* = .046), with no difference in high-density lipoprotein cholesterol. Systolic and diastolic blood pressure also tended to decrease but were not statistically significant (*P* = .087 and .075, respectively). All variables that were statistically significant remained so when adjusting for percent weight loss.

### Metabolic Response to MMTT

**Figure 1.**
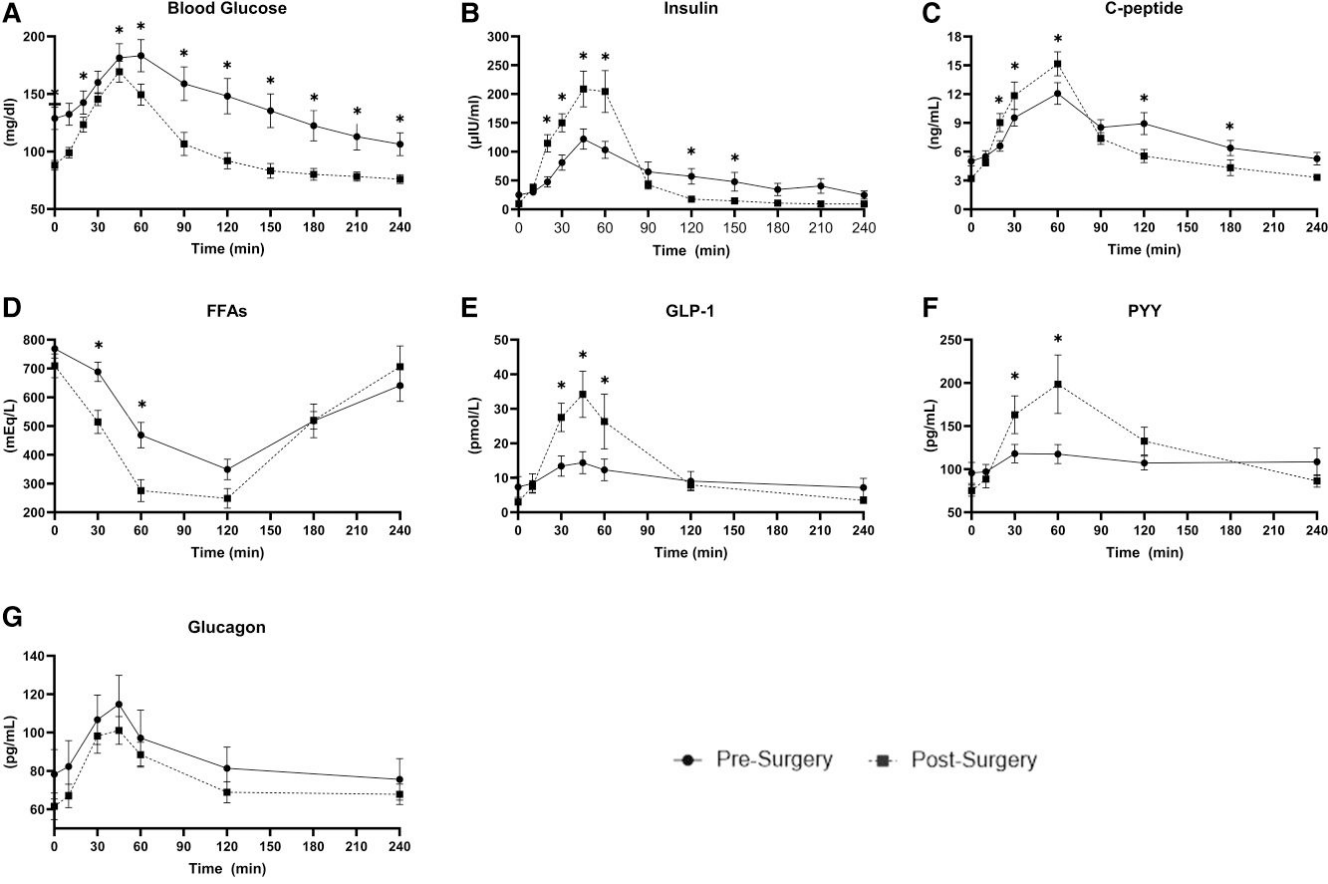
MMTT labs. MMTT results pre- and postmetabolic bariatric surgery shown as mean ± SE. Estimated marginal means were calculated for each time point of the MMTT, and pairwise comparisons were used to test whether each time point differed across visits. Significant differences at each time point are indicated by *P < .05 for comparison between pre- and post-time points in each respective lab measure. Solid circles represent presurgery and dashed squares represent postsurgery. Abbreviations: FFA, free-fatty acid; GLP-1, glucagon-like peptide 1; MMTT, mixed-meal tolerance test; PYY, peptide YY.

As measured by the MMTT, glucose, insulin, C-peptide, FFA, GLP-1, PYY, and glucagon excursions all changed significantly after surgery (Fig. 1). Fasting and postprandial blood glucose was lower across the entire MMTT study following surgery and was significantly lower at time points 0, 10, 45, 60, 90, 120, 150, 180, 210, and 240 minutes (*P* < .01 for all). Insulin secretion was significantly higher at 20, 30, 45, and 60 minutes (*P* < .001 for all) and was significantly lower at 120 and 150 minutes (*P* < .05 for both). Similar to insulin, C-peptide was significantly higher at 20, 30, and 60 minutes (*P* < .05 for all) and significantly lower at 120 and 180 minutes (*P* < .05 for both). FFAs were significantly lower at 30 and 60 minutes (*P* < .05 for both). The incretins GLP-1 and PYY were both significantly higher at 30, 45, and 60 minutes (*P* < .005 for both). Fasting fat oxidation increased (*P* = .042) with a parallel trend in reduced fasting carbohydrate oxidation (*P* = .083) and fasting RQ (*P* = .079). REE was significantly lower postsurgery (*P* < .001) (Table 3). The significant changes remained significant when adjusting for weight loss. There were no significant differences in glucagon, indirect calorimetry-assessed metabolic flexibility, or fed measures at this early 3-month postoperative time point.

**Table 3.** Indirect calorimetry

Variable	Presurgery	Postsurgery	*P* (unadjusted)	*P* (adjusted)
Time 0 VO_2_ (mL/kg/min)	2.4 ± 0.2	2.5 ± 0.2	.356	.356
Time 60 VO_2_ (mL/kg/min)	2.5 ± 0.2	2.8 ± 0.2	.251	.249
Time 0 RQ	0.84 ± 0.01	0.81 ± 0.02	.079	.076
Time 60 RQ	0.86 ± 0.01	0.85 ± 0.01	.499	.494
Time 0 REE	2199 ± 119	1733 ± 123	<.001	<.001
Time 60 REE	2262 ± 117	1882 ± 118	<.001	<.001
Time 0 Fat oxidation	0.61 ± 0.06	0.74 ± 0.06	.042	.041
Time 60 Fat oxidation	0.55 ± 0.07	0.63 ± 0.07	.305	.306
Time 0 carbohydrate oxidation	1.48 ± 0.17	1.09 ± 0.18	.083	.079
Time 60 carbohydrate oxidation	1.77 ± 0.17	1.67 ± 0.18	.604	.602
Metabolic flexibility (+60 minutes RQ—0 minutes RQ)	0.02 ± 0.01	0.04 ± 0.01	.284	.269

Continuous variables are shown as mean ± SE and were tested using repeated measures mixed-effects models and are shown for both unadjusted and adjusting for percent weight loss at 3 months. Significance was set at *P* < .05.

Abbreviations: REE, resting energy expenditure; RQ, respiratory quotient.

### Insulin Sensitivity and Secretion Dynamics

Fasting SI measured by HOMA-IR significantly improved from 8.3 ± 1.5 to 2.2 ± 1.5 (*P* = .009), as did SI by Matsuda Index during the MMTT (*P* = .048). Static and dynamic SI and efficiency were unchanged when assessed by OMM (Table 4). Insulin secretion measured by the molar-concentration of insulin from the ISR profile showed no change in timing of the peak (43.9 vs 41.9 minutes, *P* = .841), but the ISR peak heights increased by 2.5-fold during the first 30 minutes (*P* < .001), doubled during the first 60 minutes (*P* < .001) of the MMTT, and tended to increase over the entire 4-hour MMTT (*P* = .09). -Thirtyminute β-cell responsivity was unchanged, as was 60 minute, but 4-hour significantly improved (data not shown). Disposition index significantly improved from 0.14 ± 0.1 to 0.39 ± 0.1 (*P* = .027).

**Table 4.** Measures of insulin sensitivity and secretion

Variable	Presurgery	Postsurgery	*P* (unadjusted)	*P* (adjusted)
HOMA-IR	8.3 ± 1.5	2.2 ± 1.5	.009	.009
Matsuda Index	2.7 ± 0.4	3.5 ± 0.4	.048	.048
DI	0.14 ± 0.1	0.39 ± 0.1	.027	.027
Model-based variables				
Static SI	6.01 × 10^−4^	4.14 × 10^−4^	.099	.099
Dynamic SI	1.32 × 10^−4^	1.14 × 10^−4^	.45	.45
Efficiency	0.262	0.296	.293	.293
ISR 30 minutes AUC	2747	6809	<.001	<.001
ISR 60 minutes AUC	8078	16867	<.001	<.001
ISR 4 hours AUC	15439	20259	.09	.09
ISR time to peak	43.9	41.9	.841	.841
30-minute β-cell responsivity	8.58	9.59	.532	.532

Continuous variables are shown as mean ± SE and were tested using repeated measures mixed-effects models and are shown for both unadjusted and adjusting for percent weight loss at 3 months. Significance was set at *P* < .05.

Abbreviations: AUC, area under the curve; DI, Disposition Index; HOMA-IR, Homeostasis Model of Insulin Sensitivity; ISR, insulin secretion rate; SI, insulin sensitivity.

### Adverse Events

There were no significant immediate postoperative complications; however, 1 participant underwent laparoscopic cholecystectomy approximately 2 months after surgery for symptomatic cholelithiasis. No participant experienced a blood sugar under 85 mg/dL during the MMTT postsurgery or hypoglycemia at home or during the MMTT.

## Discussion

To our knowledge, IMPROVE-T2D is the first study to examine glucose and insulin homeostasis, body composition, cardiovascular risk markers, REE and nutrient oxidation, or incretin response in youth with type 2 diabetes undergoing VSG, and to do so in a prospective design. We observed notable improvements in body weight, body composition, liver transaminases as a marker of metabolic dysfunction-associated steatotic liver disease, glycemic control, fasting glucose, SI, fat oxidation, heart rate, and fasting lipid profile. Moreover, we observed markedly higher early incretin, insulin, and C-peptide secretion during the MMTT, leading to notably improved glucose tolerance and FFA suppression, and faster recovery of glucose, insulin, and C-peptide secretion back to fasting values. These changes remained significant even when statistically controlling for weight loss, implying early, weight loss independent effects of VSG in youth-onset type 2 diabetes. Importantly, these metabolic improvements occurred despite the discontinuation of diabetes medications in most participants.

MBS significantly reduces weight in youth up to 10 years [38] postoperatively, but less is known about MBS's earlier effects, and specifically regarding effects of VSG, now the most common MBS procedure worldwide [19]. The ∼19% weight loss in our cohort is slightly more than the 1% to 17% previously reported in youth at 3 months [39-41]; however, prior studies were retrospective, and all included RYGB. While we observed significant body fat reductions (14.1 kg), we also saw reductions in metabolically active FFM (4.5 kg) and thus REE, potentially increasing the risk for weight regain. In a recent meta-analysis, Nuijten et al reported >8 kg of FFM loss within 1-year post-MBS in adults, with about 55% (4.4 kg) lost within the first 3 months. While nearly identical to our findings at 3 months, the meta-analysis included adults without diabetes [42], emphasizing the importance of longer-term follow-up in youth and interventions to reduce FFM loss with MBS.

The improvement in HbA1c is notable, particularly because most participants were in the normal or prediabetes (≤6%) range while off all diabetes medications. Teen-LABS also demonstrated remission in ∼95% of youth with preexisting diabetes at 3 years postoperatively [18] and 55% at 10 years [38]. However, Teen-LABS did not include a time point before 6 months and did not focus on VSG, and thus IMPROVE-T2D adds considerably to our understanding of early metabolic changes. This early improvement could be explained by factors such as postoperative enhancements in insulin secretion, improved insulin sensitivity, decreased appetite resulting in reduced carbohydrate consumption [43], and/or changes in gut microbiome or bile acid metabolism. However, at the 3-month time point we studied, all participants were back on a normal, solid-food diet. Therefore, by the 3-month study visit, their diet is more representative of a normal composition and reflects typical eating patterns, rather than being limited to liquid meals.

Indeed, we show significant increases in insulin secretion, fasting and Matsuda Index-calculated SI, and GLP-1 and PYY following food ingestion. Peak ISR was 2 to 2.5-fold higher and peak GLP-1 and PYY secretion nearly 2.5 and 2-fold higher, respectively, postsurgery, resulting in marked reductions in blood glucose throughout the MMTT, with a return to fasting glucose levels nearly 2 hours earlier postoperatively. Although insulin secretion was significantly higher from minutes 20 to 60 postoperatively, it returned to basal levels approximately 2 hours earlier, limiting systemic exposure to hyperinsulinemia and hyperglycemia. Model-derived ISRs corroborate the large increase in insulin secretion postsurgery, specifically the first-phase insulin response. A major physiological change that occurs after MBS is accelerated gastric emptying, which may play a role in the increases seen in insulin, GLP-1, and PYY. Although gastric emptying was not measured directly in our study, the time to peak of the model-derived ISR was not different at 3 months, suggesting that gastric emptying was unlikely to be the cause of these changes. We used a modified MMTT that has previously been used in bariatric surgery research to prevent gastrointestinal distress from drinking quickly after undergoing surgery [44]. Although the drink is slowly consumed over 30 minutes, it was used in the same manner at both study visits, therefore preventing any unanticipated outcomes as it relates to ingestion such as gastric emptying or glucose/insulin dynamics. The MMTT kinetics therefore may not be directly comparable to other MMTT or OGTT studies in which the drink is consumed more rapidly; however, the glucose and insulin peaks follow similar patterns as expected upon consumption. Post-VSG, we saw a clear improvement in fasting SI as indicated by HOMA-IR and combined fasting and postprandial SI assessed by the Matsuda Index; however, there was no improvement in SI as estimated using OMM. This could be attributed to an interaction between changes in SI and insulin secretion, to Matsuda being more strongly influenced by fasting values, thus aligning more with HOMA-IR, or could be limited by our sample size, or by confounding improvements in SI at baseline from insulin-sensitizing medications, which were then discontinued. However, we also did not see significant improvements in SI 3 months post-RYGB in adolescents without diabetes using an intravenous glucose tolerance test [45] but did show significant improvement at 12 months. Some metabolic changes are likely dynamic and may relate to weight loss; thus further follow-up of our cohort is needed for final determinations.

We also documented improvements in other hormones and metabolites that play important roles in individuals with type 2 diabetes. FFA elevations correlate with muscle insulin resistance and vascular dysfunction [46]. FFA suppression improved postsurgery, suggesting improved adipose tissue SI and/or a response to the increased first-phase insulin secretion. The incretins GLP-1 and PYY both increased postoperatively. PYY and GLP-1 both induce satiety [47] and may explain the weight loss we observed with VSG. In addition, PYY may also improve islet secretory function post-MBS [48], and GLP-1 is known to increase glucose-dependent insulin secretion and suppress glucagon during hyperglycemia [49], all factors that likely contribute to the metabolic improvements seen in participants of IMPROVE-T2D. GLP-1RA use was limited to short-acting daily forms, which were withdrawn for the baseline visits to limit the possibility of GLP-1 pharmacologically increasing at baseline and were not restarted after surgery. However, a pharmacologic increase in GLP-1 at baseline would have biased us toward the null, whereas we did find significantly higher levels postsurgery, strengthening our findings. While insulin secretion was clearly increased, we saw numerical but not statistically significant changes in glucagon. The literature on glucagon changes with MBS have been mixed and differ by surgery type [50], and we may have been underpowered or assessed changes too early postoperatively to see changes in glucagon.

Some adult studies have shown short-term improvements following MBS in as little as 2 weeks postoperatively including fasting glucose, insulin, and GLP-1 and improved SI in people without diabetes [51]. However, few adult studies have investigated the response to a MMTT 3 months post-MBS. Peterli et al [52] studied 27 adults (13 RYGB and 14 VSG) 3 months post-MBS and, similar to our study, found improvements in MMTT-assessed glucose homeostasis and significantly increased postprandial insulin, GLP-1, and PYY, with improvements in HOMA-IR. However, only 3 of the 14 participants who underwent VSG had type 2 diabetes. Moreover, many prior MMTT studies have measured only total secretion via area under the curve, whereas our mixed-effects models improve time point-specific and/or kinetic assessments of the curve. Overall, it appears that adults and youth with type 2 diabetes undergoing VSG both improve multiple metrics of diabetes health, but the extent to which they can be compared directly is limited due to study design differences.

The youth in our study also tolerated the surgical procedure well, with only 1 requiring laparoscopic cholecystectomy approximately 2 months postoperatively. Youth in general appear to tolerate VSG as well or better than adults. In a study directly comparing 5-year outcomes of VSG in youth and adults, there were no differences in postoperative complications, and none of the adolescents developed major complications, leakage, or intraperitoneal bleeding [53].

The strengths of IMPROVE-T2D include our understudied but severely affected and growing population of youth-onset type 2 diabetes and novel data on short-term hormonal effects that occur prior to full weight loss from VSG in this population. We utilized a frequently sampled, 4-hour MMTT that represents a physiological assessment and permits stimulation of key incretin responses vs an insulin clamp, intravenous glucose tolerance test, or oral glucose tolerance test due to the utilization of the gut and inclusion of fats and protein, respectively. Moreover, traditional tolerance tests are less than 4 hours and often rely on area under the curve, missing valuable time-dependent changes in metabolic outcomes. We also included multiple methods for assessing SI and insulin secretion and specifically adapted our gold-standard OMM methods to our highly insulin resistant adolescent population. In addition, all procedures were done prospectively, under standardized research conditions, including control of activity, diet and medication timing, equal distribution of males and females, requiring diabetes antibodies to exclude type 1 diabetes, and including well-phenotyped participants. Study weaknesses include the small sample size, which was not powered to look at sex differences, and lack of a control group without diabetes, which may affect the generalizability to other cohorts and the ability to detect differences in some hormones. We also lacked measurement of dietary intake or physical activity; however, dietary assessment in this population is known to be inaccurate with existing methods due to recall bias and underreporting [54]. Caution is also warranted when interpreting insulin sensitivity changes derived from OMM or the Matsuda Index in this study, as these indices were originally validated using methodologies different from those employed here, such as oral glucose tolerance tests or alternative meals consumed in a single, shorter time frame. The MMTT protocol in our study, consumed over 30 minutes, may not be directly comparable to other studies using different beverages or protocols. However, these calculations were adapted to facilitate within-participant comparisons pre- and postsurgery and modified to accommodate the postsurgical needs of our study population. Further, our short-term outcomes may not reflect long-term improvements, thus we are continuing to follow this cohort to prospectively study longer-term outcomes and durability.

In summary, we present the first study showing early MMTT outcomes in youth-onset type 2 diabetes undergoing VSG. VSG was well-tolerated in this age group, and within 3 months, VSG induced notable improvements in weight, body composition, SI and insulin secretion, glucose tolerance, FFA suppression and fat oxidation, glycemic control, and cardiometabolic health, including diabetes remission in the majority, supporting consideration of VSG for weight and metabolic management in this high-risk population. These changes remained statistically significant when adjusting for weight loss, which may suggest weight loss independent effects, potentially implicating direct surgical effects in youth-onset type 2 diabetes such as incretin responses, arguing for more pediatric studies of GLP-1 and other incretin medications. Future studies will evaluate longer-term durability of these improvements at 1 year, which will allow for further analyses to compare this early time point with the 1-year mark and identify potential predictors of longer-term outcomes that could indicate who is at risk for failure, needing earlier additional intervention. Further investigation of the underlying mechanisms including hyperglycemic clamp methods to further assess intravenously stimulated SI and insulin secretion, magnetic resonance imaging to precisely evaluate fat distribution in specific abdominal and cardiac depots and cardiac function, and assessment of relationships between FFM loss and health outcomes over time.

## Data Availability

Some or all datasets generated during and/or analyzed during the current study are not publicly available but are available from the corresponding author on reasonable request.

## Clinical Trials Registration

NCT03620773
